# Magnitude or Multitude – What Counts?

**DOI:** 10.3389/fpsyg.2018.00522

**Published:** 2018-04-12

**Authors:** Martin Lachmair, Susana Ruiz Fernández, Korbinian Moeller, Hans-Christoph Nuerk, Barbara Kaup

**Affiliations:** ^1^Leibniz-Institut für Wissensmedien, Tübingen, Germany; ^2^FOM-Hochschule für Oekonomie und Management, Essen, Germany; ^3^LEAD Graduate School and Research Network, University of Tübingen, Tübingen, Germany; ^4^Department of Psychology, University of Tübingen, Tübingen, Germany

**Keywords:** numerical cognition, grammatical number, space-number associations, space-word associations, grounded cognition

## Abstract

Recent studies revealed an association of low or high numbers (e.g., 1 vs. 9) and word semantics referring to entities typically found in upper or lower space (e.g., *roof* vs. *root*) indicating overlapping spatial representations. Another line of research revealed a similar association of grammatical number as a syntactic aspect of language and physical space: singular words were associated with left and plural words with right - resembling spatial-numerical associations of low numbers with left and high numbers with right.

The present study aimed at integrating these lines of research by evaluating both types of spatial relations in one experiment. In a lexical decision task, pairs of a numerical cue and a subsequent plural noun were presented. For word with spatial associations (e.g., *roofs* vs. *roots*) number magnitude was expected to serve as a spatial cue. For spatially neutral words (e.g., *tables*) numbers were expected to cue multitude. Results showed the expected congruency-effect between the numbers and words with spatial associations (i.e., small numbers facilitate responses to down-words and high numbers to up-words). However, no effect was found for numbers and spatially neutral words. This seems to indicate that spatial aspects of word meaning may be related more closely to the magnitude of numbers than grammatical number is to the multitude reflected by numbers – at least in the current experimental setting, where only plural words were presented.

## Introduction

Human language and human’s ability for numerical cognition evolved in the context of the physical conditions on Earth. For example, gravitational force of earth gives us an omnipresent reference of vertical space. Thus, it may come with no surprise that such conditions have shaped human cognitive systems. This, for example, is reflected in human language, which is full of words and phrases that explicitly or implicitly express spatial attributes related to the vertical spatial dimension (cf. Levinson, 2003; Lakoff and Johnson, 2008). In addition, this vertical spatial dimension also plays an important role in numerical cognition (cf. Dehaene, 2011; Fischer and Shaki, 2014 for a review). In cognitive science, important lines of research pursue how information that is captured in such symbolic systems like language and numbers is represented mentally. In principle, it is possible that such representations are based on abstract, arbitrary and amodal cognitive processes (e.g., Fodor, 1975) that reside within memory systems separate from the brain’s modal systems (e.g., perception, action; Tulving, 1972). However, over the last decades there has been accumulating evidence for mental representations based on sensorimotor experience, suggesting an important role of sensorimotor aspects in knowledge representations (cf. Barsalou, 2012).

### Spatial Representations as a Common Ground of Words and Numbers

Several studies showed that words may automatically activate spatial information related to the typical location of their referents. For example, a word like “roof” whose referent is typically located and experienced in upper vertical space shifts attention upwards. In contrast, a word like “root” whose typical location is in lower vertical space was observed to shift attention downwards (e.g., Lachmair et al., 2011; Dudschig et al., 2013; Thornton et al., 2013).

For the case of numbers, their dominant and most ubiquitous spatial association is typically referred to by the metaphor of a horizontal mental number line (e.g., Restle, 1970; Dehaene et al., 1993) on which numbers are represented according to their magnitude from left to right (cf. Fischer and Shaki, 2014, for a review). However, many authors considered this unidimensional metaphor as insufficient (e.g., Dehaene, 2011; Cipora et al., 2015; Winter et al., 2015). Interestingly, for such directional spatial-numerical associations, in which a certain direction in space is associated with larger numbers (i.e., right, up, etc.), different dimensions may play a role. For instance, there are also findings suggesting a vertical representation of numbers from lower (small numbers) to upper vertical space (larger numbers; e.g., Schwarz and Keus, 2004). Given that both dimensions are associated with number magnitude, the question arose which spatial dimension (i.e., horizontal or vertical) may be associated more strongly with the representation of number magnitude (cf. Holmes and Lourenco, 2012). In fact, Fischer and Brugger (2011) suggested a hierarchical view of spatial-numerical associations differentiating grounded, embodied and situated aspects in the mental representation of numbers (see also Myachykov et al., 2014). According to this view, the metaphor of a horizontal mental number line is driven by cultural conventions, practices and habits (e.g., left-to-right reading direction) and is therefore considered embodied. In contrast, the vertical representation of numbers was proposed to be grounded in the sense that it is based on and reflects universal physical conditions like gravitational force of earth (Fischer and Brugger, 2011) – and thus be more general than the embodied metaphor of a mental number line. This may be illustrated easily by considering the example of filling a glass with water. As one pours more water into the glass the surface level of the water in the glass rises. This reflects a general grounding experience of more of something (in this case water) being associated spatially and accordingly numerically higher magnitudes regardless of culture or place on earth (cf. Lachmair et al., 2017).

A recent study investigated these strong spatial relationships using numbers and nouns. Lachmair et al. (2014) hypothesized that there may be a common or overlapping representational space for the domains of numbers and words referring to entities typically located in upper or lower vertical space. And indeed, they observed that processing low and high numbers, respectively, affected the processing of subsequent words referring to objects with a typical location in lower or upper vertical space (henceforth referred to as down- and up-words, respectively). In particular, the authors found shorter reaction times in a lexical decision task for a congruent combination of low number primes (e.g., “1,” “2”) and subsequently presented down-words (e.g., “floor”) and high number primes (e.g., “8,” “9”) and subsequently presented up-words (e.g., “sky”). In contrast, reaction times were longer in incongruent combinations of numbers and words (i.e., combinations of low number primes followed by up-words and high number primes followed by down-words). The authors interpreted these results as evidence for an overlap in the meaning representations of numbers and words referring to entities with a typical location in upper vs. lower vertical space (Lachmair et al., 2014). This overlap presumably results from the fact that similar mental states are being activated when interacting with the referents of these two types of symbols in the world (cf. Barsalou, 2012). Thus, according to the above mentioned view proposed by Fischer and Brugger (2011), one may conclude that similar to the grounding of number magnitude on vertical space, attentional shifts subsequent to processing words like “sky” or “floor” also reflect effects of groundedness, because their mental representations integrate experiences according to omnipresent physical conditions.

### Embodied or Grounded Spatial Representation of Grammatical Number

Beyond commonalities with respect to spatial attributes of word meaning, words and numbers are also interrelated by the syntactical concept of grammatical number. A recent study by Roettger and Domahs (2015) showed that the flexion of German nouns expressing the multitude of their referent(s) also has a spatial association. The authors found a horizontal spatial association, indicated by faster reaction times for singular words when responded to with the left compared to the right hand, whereas a reversed pattern was observed for words in plural form. Although this pattern was found in relatively late stages of the response process and seemed to vary with the complexity of stimulus decoding, this result indicates that *multitude* derived from the syntactic concept of grammatical number is represented on a horizontal axis with lower quantities (i.e., singular) associated with left and higher quantities (i.e., plural) associated with right.

This raised the question at which level grammatical number and physical space interact. According to the hierarchical structure proposed by Fischer and Brugger (2011, see also Myachykov et al., 2014; Lachmair et al., 2017), the horizontal spatial representation of grammatical number may be considered *embodied* because it relies on an overlearned cultural convention and not on an omnipresent physical law that may shape human cognition.

However, there is also evidence suggesting a grounded origin of the spatial representation of multitude. In their study, Berent et al. (2005) argued that readers extract syntactic grammatical number of bare nouns automatically and represent it in a way that is comparable to the representation of number they extract from visual stimuli. Thus, one might conclude that identifying quantities is a fundamental and universal ability of the human visuo-spatial perceptual system (Anobile et al., 2016). In turn, this would imply the concept of syntactic grammatical number to be grounded. However, even though this fundamental ability may be invariant across cultures, one may doubt that it is deeply associated with mental representations of grammatical number. If so, one would expect a universal cross-cultural representational system of grammatical number. However, this is obviously not the case when considering languages that differentiate explicitly between singular and plural like English or German on the one and languages that have very little singular/plural marking like Mandarin or Japanese on the other hand (e.g., Downing, 1996; Sarnecka, 2014; see Overmann, 2015 for an overview) or special cases such as some Slavic languages in which grammatical number differs for different number ranges (e.g., Polish). Thus, it is unclear how the mental representation of grammatical number may be embedded in a hierarchical structure as proposed by Fischer and Brugger (2011). It is however, well conceivable that the mental representation of grammatical number is grounded and embodied (and maybe even situated). Which representation is actually accessed may depend on the dimension (horizontal or vertical) in which the relation between grammatical number and numerical magnitude is examined.

### Magnitude Versus Multitude

Against this background, the question arises which representation of meaning is affected, multitude or magnitude, when nouns denoting objects in vertical space are presented in plural subsequent to numerical cues. Following the hierarchical view of Fischer and Brugger (2011) one would assume that grounded effects override embodied effects in the vertical dimension, which means a grounded effect should prevail.

In the present study, we aimed at evaluating this hypothesis. We presented nouns referring to objects typically located in lower vs. upper vertical space (e.g., “worms” vs. “birds”) and spatially neutral nouns (e.g., “machines”) in plural form after either a low or high number prime. Put differently, our words were congruent or incongruent with the number cues with respect to two different dimensions. With respect to their semantics, up-words are congruent with high numbers and incongruent with low numbers whereas down-words are congruent with low numbers and incongruent with high numbers. With respect to their grammatical number, up- and down-words are both congruent with high numbers and incongruent with low numbers. Neutral plural nouns, in contrast are only congruent or incongruent with respect to one dimension, namely grammatical number; they are congruent with high number cues and incongruent with low number cues (see **Table 1**). In our study, we were interested in evaluating the relative impact of congruency on the two dimensions.

**Table 1:** Congruency of numbers and words according to word meaning or grammatical number. “+” denotes congruency,“-” denotes incongruency and “°” neither congruency nor incongruency.

	word meaning				grammatical number		
	Up	Down	Neutral		Up	Down	Neutral
2, 3	-	+	°		-	-	-
8, 9	+	-	°		+	+	+

Against the above described background, our hypotheses were as follows. According to Fischer and Brugger (2011), the representation of number magnitude is assumed to be grounded in the vertical spatial dimension whereas the representation of syntactic grammatical multitude is assumed to be embodied on a horizontal spatial dimension (Roettger and Domahs, 2015). As such (i) due to their grounding on vertical space, an effect of congruency for numerical cues and word meaning associated with lower and upper space should be observed with faster reaction times for congruent number-word pairs (low numbers/down-words, high numbers/up-words) compared to incongruent pairs (low numbers/up-words, high numbers/down-words, cf. Lachmair et al., 2014). Moreover, this line of argument would suggest (ii) an embodied effect of numerical cues on spatially neutral words due to their plural word form with faster reaction times for high numbers compared to low numbers as shown in Roettger and Domahs (2015). However note, due to the hierarchical superiority of grounded over embodied effects, it is also possible that grounded influences may be processed predominantly by definition. As such, the preference of grounded effects may generally reduce the probability to observe embodied effects such as (ii). Importantly, because of the different nature of the two potential influences (grounded vs. embodied) there should be (iii) no interaction between the two. In other words, the congruency effect in (i) should not be affected by congruency with respect to grammatical number. There is, thus, no reason to expect that the congruency effect between numerical cues and word meaning will differ between up- and down-words. We will refer to this hypothesis as the Grounded-Embodied-Hypothesis in the following.

However, following Berent et al. (2005) both representations of numerical magnitude and syntactic grammatical multitude are grounded. This hypothesis predicts (i) and (ii) as above but without the possibility of (ii) being overridden by (i). Importantly, in contrast to the above discussed Grounded-Embodied-Hypothesis, this hypothesis would predict congruency effects to differ between up- and down-words. In particular, for up words, where congruency with respect to word meaning and congruency with respect to grammatical number fall together, a larger overall congruency effect is to be expected. Contrarily, for down-words incongruence on the two dimensions should result in a smaller overall congruency effect. We will refer to this hypothesis as the Grounded-Grounded-Hypothesis in the following.

## Materials and Methods

Participants performed a lexical decision task on plural nouns denoting objects that are typically encountered in the upper or lower vertical space, as well as spatially neutral words (e.g., *roofs* vs. *roots* vs. *machines*, respectively). These nouns were preceded by either small (2, 3) or large number cues (8, 9). Please note, the study by Lachmair et al. (2014) investigated priming effects of numbers “1,” “2,” “8,” and “9” on words in singular word form. However, using “1” as a cue might lead to conflicts when processing the plural word form employed in this study. Therefore, the number cue “1” was replaced by the number cue “3,” so that all number cues denoted plurality and would not interfere with our study goals.

### Participants

Twenty-two right-handed native speakers of German (17 female; *M*_age_ = 22.64 years, *SD* = 3.17) took part in this experiment. Experimental testing was in agreement with the guidelines for good scientific practice at the University of Tübingen (Germany). Participants’ anonymity was always preserved. All participants gave their written informed consent and received course credit or financial reimbursement of 8 Euros per hour for participation. All participants had normal or corrected-to-normal vision.

### Materials and Apparatus

Materials consisted of the numbers *2*, *3*, *8*, and *9*, as well as 60 German nouns and 20 pseudo words. Of the 60 nouns, 20 referred to an object that is typically located in upper vertical space, 20 referred to objects that are typically located in lower vertical space and 20 referred to objects denoting a neutral position according to verticality. All nouns were taken from the study by Lachmair et al. (2011), being controlled for frequency, length and for the typical vertical position of their referent (cf. Lachmair et al., 2011). Words and numbers were presented in white against a black background on a 17” CRT monitor. The vertical visual angle varied according to word length between 2.15° and 5.4°. Responses were recorded using a standard QWERTZ keyboard with horizontally aligned response keys. We employed the ‘y’-key for left hand responses and the ‘-’-key for right hand responses.

### Procedure and Design

Participants were presented with plural nouns preceded by a one-digit number prime (i.e., 2, 3, 8, or 9). Primes and subsequent nouns were presented in the center of the screen. Participants had to decide whether the presented letter string was a correct German word or not. Each participant started with a short practice block (32 trials) consisting of words of the word-categories UP, DOWN, NEUTRAL and PSEUDOWORDS presented subsequent to numerical cues. Then, in the first half of the experiment, participants had to respond with a left key press to words and a right key press to pseudo-words. With another 32 practice trials the second half of the experiment started in which hand-to-response mapping was reversed. Each trial started with a centered fixation cross (500 ms), followed by a number prime presented for 300 ms. Then the (pseudo)-word appeared and stayed on the screen until a response occurred.

Response times (RTs) were measured as the time from word onset to a key response. Each stimulus was presented eight times (four times in each half), resulting in a total of 640 experimental trials (480 word-trials and 160 pseudo word-trials), subdivided into 8 blocks, separated by self-paced breaks with error information. Each experimental half started with a short practice block. The design was a 2×3 design with the numerical magnitude of the number cues (low, high) and the implicit locational association of words (word category: up, down, neutral) as within-participant factors. Please note, that the locations of the response keys to the left and to the right were not important for the design, because their spatial alignment was horizontal, not vertical and the mapping with pseudo and non-pseudo words was counterbalanced.

## Results

**FIGURE 1:**
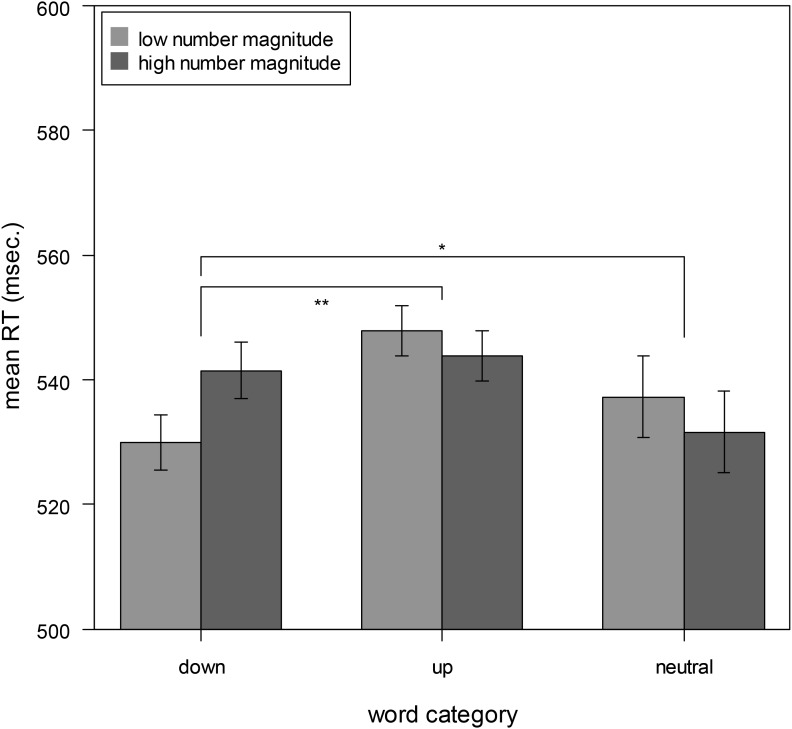
Mean reaction times as a function of implicit locational association of words (up, down, neutral) and numbers (high, low). Error bars represent 95% confidence intervals. ^∗^*p* < 0.05; ^∗∗^*p* < 0.01.

All data were analyzed using R (R Development Core Team, 2017). The data of one participant had to be excluded due to an error rate exceeding 20%. Responses to pseudo words were excluded from analyses. A trimming procedure further eliminated responses slower than 200 ms (0.03%), erroneous responses (2.54%), as well as responses for which RT deviated by more than 3 SDs from the individual’s mean in the respective condition. This led to an additional loss of 1.91% of the data. The means of the remaining reaction times are displayed in **Figure 1** as a function of word category and number cue magnitude. For investigating our hypotheses, we conducted a 2×3 ANOVA with the within-factors number cue magnitude (low vs. high) and word category (up vs. down vs. neutral).

The ANOVA revealed a significant main effect of word category [*F*(2,40) = 4.21, *p* = 0.022, $\eta_{p}^{2}$ = 0.17] indicating slower responses to up-words (*RT*_mean_ = 546 ms, *SD* = 136 ms) compared to down- (*RT*_mean_ = 536 ms, *SD* = 131 ms) and neutral-words (*RT*_mean_ = 534 ms, *SD* = 126 ms). Additionally, there was a significant interaction between number cue magnitude and word category [*F*(2,40) = 4.40, *p* = 0.019, $\eta_{p}^{2}$ = 0.18]. To break down this 2×3 interaction, we conducted several additional analyses.

First, we excluded the neutral words and conducted a 2 (number cue magnitude: low vs. high) × 2 (word category: up vs. down) ANOVA which revealed a significant two way interaction [*F*(1,20) = 8.35, *p* = 0.009, $\eta_{p}^{2}$ = 0.29]. As can be seen from inspecting the means in **Figure 1**, reaction times were shorter in congruent conditions (i.e., high numbers followed by up-words and low numbers followed by down-words) compared to incongruent conditions (i.e., low numbers followed by up-words and high numbers followed by down-words), and the difference between the congruent and the incongruent condition was numerically larger for down-words than for up-words.

Second, we excluded the up-words and conducted a 2 (number cue magnitude: low vs. high) × 2 (word category: down vs. neutral) ANOVA. This ANOVA also revealed a significant two-way interaction [*F*(1,20) = 5.8, *p* = 0.026, $\eta_{p}^{2}$ = 0.23]. Again, reaction times in the congruent condition were shorter than those in the incongruent condition, with the difference being numerically larger for down words than for neutral words.

Third, we excluded the down-words and conducted a 2 (number cue magnitude: low vs. high) × 2 (word category: up vs. neutral). This ANOVA did not show a significant interaction effect (*F* < 1, *p* = 0.81).

Finally, evaluating simple effects *t*-tests revealed for down-words significantly faster RTs when they followed a low number cue (*RT*_mean_ = 530 ms, *SD* = 50 ms) compared to a high number cue (*RT*_mean_ = 542 ms, *SD* = 57 ms; *t*(20) = 3.16, *p* = 0.005, $\eta_{p}^{2}$ = 0.33). However, for up-words, a *t*-test indicated no significant advantage of RTs when they were presented following a high number cue (*RT*_mean_ = 544 ms, *SD* = 64 ms) as compared to a low-number cue (*RT*_mean_ = 548 ms, *SD* = 62 ms; *t*(20) = -1.44, *p* = 0.16, $\eta_{p}^{2}$ = 0.09). A similar finding was obtained for neutral words for which RTs did not differ significantly following a low (*RT*_mean_ = 537 ms, *SD* = 61 ms) or high number cue (*RT*_mean_ = 532 ms, *SD* = 56 ms; *t*(20) = -0.96, *p* = 0.35, $\eta_{p}^{2}$ = 0.04, see **Figure 1**).

## Discussion

Recent research indicated spatial associations for words referring to entities with a typical location in vertical space, as well as for numbers. In the current study, we were interested in the interrelation between these spatial associations. Specific attention was paid to the role played by the magnitude and multitude status of the words. Participants were presented with a numerical cue (low: 2, 3 vs. high: 8, 9) and a subsequent word in plural flexion. These words were nouns that referred to entities typically located in lower or upper vertical space (e.g., roots vs. roofs) or spatially neutral nouns (e.g., “tables”). Considering the idea of a grounding of numbers and word meanings in vertical space, we evaluated whether the congruency between number magnitude and spatial aspects of word meaning generalizes to plural word forms, and if so how this effect is affected by grammatical number.

Accordingly, two hypotheses were formulated. The Grounded-Embodied-Hypothesis predicts (i) a congruency between numerical cues and word meanings associated with lower and upper space according to their grounding in vertical space, and (ii) an embodied effect of numerical cues on spatially neutral words due to their plural word form. However, according to Fischer and Brugger (2011) the grounded effect of (i) may also override the embodied effect of (ii) causing the latter not to show.

In contrast, the Grounded-Grounded-Hypothesis would predict a more robust influence of numerical cues on spatially neutral words due to their plural word form which should not be overridden by spatial congruency of numbers and up- and down words. In addition, an influence of grammatical number on the congruency effect between number magnitude and spatial cues conveyed by word meaning would be expected. In particular, a larger congruency effect should be observed for up-words than for down words (see above).

Our results substantiated the Grounded-Embodied-Hypothesis: First, we observed a significant interaction between the magnitude of numerical cues and the word meaning of up vs. down words. We observed faster reaction times for congruent number-word pairs (high number/up-word, low number/down-word) compared to incongruent number-word pairs (high number/down-word, low number/up-word). Second, it appears that the difference of reaction times between low and high number cues was more pronounced for down-words than for up-words, which is opposite to what was expected from the Grounded-Grounded-Hypothesis.

Moreover, given that no congruency effect was observed for syntactic grammatical number for the neutral words, one might conclude that a spatial mapping for multitude as suggested by Roettger and Domahs (2015) may not have been sufficiently activated. This claim is further corroborated by additional analyses more closely reflecting analyses and results of Roettger and Domahs (2015) who primarily observed the congruency effect in late processing stages. When we only considered reaction times larger than the median of each participant for neutral words following high or low number cues, this did not reveal any indication of a congruency effect according to grammatical number for neutral words either.

In our view, there exist two possible, not mutually exclusive explanations for this pattern of results, which is in contrast to the study by Roettger and Domahs (2015).

First, we focused on the vertical dimension, while Röttger and Domahs focused on the horizontal one. As we laid out in the introduction, directional spatial-numerical associations in the vertical dimension are assumed to be more grounded, while horizontal ones are thought to be more embodied. Because directional associations of numbers and space are related to reading direction (which is an embodied experience), it is conceivable that grammatical number as a language attribute may also be more prone to embodied influences. However, as embodied influences are weaker in the vertical condition, these might not have been sufficient to automatically activate a directional association of multitude (grammatical number) with number magnitude.

Second, we presented participants only with plural nouns and not with singular and plural words in one experiment. This might have decreased the saliency of grammatical number in contrast to Roettger and Domahs (2015) in two ways. First, because there is no variation of singular and plural, neither in the grammatical forms of the nouns, nor in the grammatical number associated with the digits (i.e., also only plural because 1 was excluded), grammatical number may not have been salient enough to influence results significantly. Second, and maybe even more importantly, multitude and grammatical number was not task-relevant. This may have also reduced saliency. However, based on the current data, we can at least infer that the activation of grammatical number may not be as automatic as suggested, for example, by Berent et al. (2005). Clearly, this issue deserves further investigation in the future.

In contrast to the lack of effects for multitude, our data suggest that co-activation of spatial attributes of number magnitude and the implicit down- and up-ward associations of up and down words are due to automatic processes resulting from the Groundedness. In turn, this led to the obtained congruency effect. Thus, considering the proposition of Fischer and Brugger (2011, see also Myachykov et al., 2014), our results substantiate the hypothesis that associations between implicit down- and up-ward attributes of word meaning and number magnitude are spatially grounded. Note, however, that in contrast to multitude, both number magnitude (small 2, 3 vs. large 8, 9) and word meaning (down-words, up-words, neutral words) was varied. This was not the case for multitude (only plural words were used), which might have played a role in the pattern of results obtained, even though both number magnitude and word meaning were also not task-relevant in our lexical decision task.

Interestingly, these data are also consistent with the notion that linguistic influences on number processing seem to occur on different representational levels. In their recent taxonomy of linguistic influences on number processing, Dowker and Nuerk (2016) differentiate between several linguistic levels at which number processing may be influenced. For the current study, influences on the syntactic and the semantic level are most relevant because the association between numerical magnitude (low/high) and word meaning (e.g., roots/roofs) is driven by the semantics of the words. In contrast, the association of number magnitude (low/high) and grammatical number (singular/plural) refers to the syntactic attribute of grammatical number. The observed result pattern suggests the association of number magnitude and word meaning to be grounded according to the framework of Fischer and Brugger (2011). This may have prevented the observation of an association of number magnitude and syntactic grammatical number, which is considered to be embodied in the horizontal dimension (cf. Roettger and Domahs, 2015). As such, this implies that semantic and syntactic linguistic influences on number processing may not interact on the same representational level. Instead, associations at the level of the meaning of words (i.e., up- vs. down-words) and numbers (i.e., the numerosity they reflect) seem to be more prominent as compared to associations across semantic (i.e., the numerosity they reflect) and syntactic (grammatical number) levels.

In summary, the present study showed a spatial congruency between low and high number magnitude cues (e.g., 2 vs. 8) and words referring to objects up or down in the world presented in plural word form. No influence of grammatical number on spatially neutral words or on the spatial congruency effect was found. Thus, together with the results of the study by Lachmair et al. (2014) this supports the view of a grounded spatial congruency between numbers and word meaning regardless of the syntactical word form. Future research is needed to substantiate this claim and to investigate (i) whether it is a general pattern that associations are most prominent when levels of linguistic and numerical processing match or (ii) whether certain (situated) experimental conditions moderate or mediate the differences observed between associations of magnitude or multitude of numbers and word meaning.

## Author Contributions

ML, BK and H-CN: conception. ML: analysis and drafting. ML, SRF, H-CN, BK and KM: interpretation, approval, and agreement. BK, SRF, H-CN and KM: revising.

## Conflict of Interest Statement

The authors declare that the research was conducted in the absence of any commercial or financial relationships that could be construed as a potential conflict of interest. The handling Editor declared a past co-authorship with one of the authors H-CN.
